# To: Death by community-based methicillin-resistant *Staphylococcus aureus*: case report

**DOI:** 10.62675/2965-2774.20240040-en

**Published:** 2024-06-14

**Authors:** Fulvio Alexandre Scorza, Carla Alessandra Scorza, Josef Finsterer

**Affiliations:** 1 Universidade Federal de São Paulo Escola Paulista de Medicina Disciplina de Neurociência São Paulo SP Brazil Disciplina de Neurociência, Escola Paulista de Medicina, Universidade Federal de São Paulo - São Paulo (SP), Brazil.; 2 Neurology and Neurophysiology Center Department of Neurology Vienna Austria Department of Neurology, Neurology and Neurophysiology Center - Vienna, Austria.

**To the Editor**

We read with interest Vieira et al.'s article about a 13-year-old male who died of necrotizing pneumonia caused by infection with community-acquired methicillin-resistant *Staphylococcus aureus* (CA-MRSA).^(1)^ The patient was initially misdiagnosed with tonsillitis but developed pneumonia complicated by massive bronchial and pulmonary bleeding, extensive mediastinal and subcutaneous emphysema, sepsis, septic and hypovolemic shock, and disseminated intravascular coagulation with multiorgan ischemia.^(1)^ Blood culture grew oxacillin-resistant *S. aureus* and multisensitive *Haemophilus influenzae*.^(1)^ Despite extensive diagnostic and therapeutic measures, the patient died.^(1)^ Autopsy revealed that the bacterial infection had caused tissue necrosis, leading to loss of integrity of the bronchial tree and leakage of air into the tissue, as well as necrosis of the vessel walls, resulting in diffuse hemorrhage of the alveolar and lower respiratory tract.^(1)^ The case report is compelling, but some points should be discussed.

First, the patient did not undergo a detailed neurological examination, although one of the first symptoms was headache.^(1)^ After the initial cardiopulmonary arrest and asystole, it took six minutes for sinus rhythm to be restored.^(1)^ One hour after the first resuscitation, the patient developed anisocoria.^(1)^ After the second asystole and successful resuscitation, sufficient oxygen saturation could not be maintained despite mechanical ventilation at high settings.^(1)^ The patient was diagnosed with sepsis, septic shock and disseminated intravascular coagulation.^(1)^ Under these circumstances, clinical neurologic examination and cerebral imaging using magnetic resonance imaging (MRI) would have been mandatory. It is crucial to determine whether the anisocoria that occurred one hour after resuscitation was due to intracerebral hemorrhage or whether the patient had an ischemic stroke, subarachnoid bleeding, subdural hematoma, epidural hematoma, or venous sinus thrombosis. Since the patient could not achieve sufficient oxygen saturation after the second cardiopulmonary arrest one hour after the first cardiopulmonary arrest, cerebral hypoxia must be ruled out.

Second, blood cultures also revealed *H. influenzae*, which was interpreted as a coinfection. How can the authors be sure that the pneumonia, sepsis and disseminated intravascular coagulation were due to CA-MRSA and not *H. influenzae* infection? In a study of 282 children with necrotizing pneumonia, *H. influenzae* was determined to be the cause in 28 patients.^(2)^

Third, the brain autopsy findings were not reported in detail.^(1)^ It is important to determine whether there were any signs of ischemic stroke, intracerebral hemorrhage, subdural hematoma, epidural hematoma, subarachnoid bleeding, or venous sinus thrombosis. In addition, signs of septic encephalopathy, MRSA meningitis, encephalitis, or cortical or subcortical cerebral hypoxia should be reported.

Fourth, severe acute respiratory syndrome coronavirus 2 (SARS-CoV-2) infection was not ruled out. Since the event occurred during the pandemic, it would have been mandatory to report the reverse transcription polymerase chain reaction results for SARS-CoV-2. Is it possible that the pneumonia was actually caused by SARS-CoV-2 and that the presence of CA-MRSA and H. influenzae were just superinfections? There are some cases of necrotizing pneumonia caused by SARS-CoV-2 in the literature.^(3)^

Fifth, several coagulation parameters were clearly abnormal. The international normalized ratio was 3.76, the prothrombin time was 21% (n, > 70%), and the partial thromboplastin time was 163 seconds (n, 29 - 38 seconds). Were these abnormalities due to consumption coagulopathy, or did the patient have a history of coagulation disorder? Is it possible that the intrapulmonary bleeding was not caused by tissue necrosis but rather by the presumably acquired coagulation disorder?

In summary, the study has several limitations that complicate the interpretation of the results. Addressing these limitations could strengthen and reinforce the statement of the study. Before fatal necrotizing pneumonia can be attributed to CA-MRSA, alternative infectious agents must be thoroughly excluded, and neurological complications require extensive diagnostic management.
