# Supplementary material for: To: Death by community-based methicillin-resistant Staphylococcus aureus: case report
Source: Crit Care Sci. 2024 Jun 14;36:e20240040en. doi: 10.62675/2965-2774.20240040-en (PMC11208040; doi:10.62675/2965-2774.20240040-en)
Supplement: Translation

# Translation: Para: Morte por *Staphylococcus aureus* comunitário resistente à meticilina: relato de caso

**DOI:** 10.62675/2965-2774.20240040-pt

**Authors:** Fulvio Alexandre Scorza, Carla Alessandra Scorza, Josef Finsterer

**Affiliations:** 1 São Paulo SP Brasil Disciplina de Neurociência, Escola Paulista de Medicina, Universidade Federal de São Paulo - São Paulo (SP), Brasil.; 2 Viena Áustria Departamento de Neurologia, Neurology and Neurophysiology Center - Viena, Áustria.

**Ao Editor**

Lemos com interesse o artigo de Vieira et al. sobre um paciente do sexo masculino, de 13 anos, que faleceu por pneumonia necrosante causada por *Staphylococcus aureus* comunitário resistente à meticilina adquirido na comunidade (CA-MRSA - *community-acquired methicillin-resistant Staphylococcus aureus*).^(1)^ O paciente foi inicialmente diagnosticado erroneamente com amigdalite, mas desenvolveu pneumonia complicada por sangramento brônquico e pulmonar profuso, enfisema mediastinal e subcutâneo extenso, sepse, choque séptico e hipovolêmico e coagulação intravascular disseminada com isquemia multiorgânica.^(1)^ A hemocultura revelou *S. aureus* resistente à oxacilina e *Haemophilus influenzae* multissensível.^(1)^ Apesar de medidas diagnósticas e terapêuticas extensivas, o paciente faleceu.^(1)^ A autópsia revelou que a infecção bacteriana havia causado necrose tecidual, levando à perda da integridade da árvore brônquica e extravasamento de ar para os tecidos, além de necrose das paredes vasculares, desencadeando hemorragia alveolar difusa e do trato respiratório inferior.^(1)^ O relato de caso é impressionante, mas alguns pontos devem ser discutidos.

Primeiramente, o paciente não foi submetido a um exame neurológico detalhado, embora um dos primeiros sintomas tenha sido cefaleia.^(1)^ Após a primeira parada cardiorrespiratória em assistolia, levou 6 minutos para que o ritmo sinusal fosse restaurado.^(1)^ Uma hora após a primeira ressuscitação, o paciente evoluiu com anisocoria.^(1)^ Após a segunda assistolia e ressuscitação bem-sucedida, não foi possível manter a saturação de oxigênio adequada, apesar do uso de parâmetros elevados de ventilação mecânica.^(1)^ O paciente foi diagnosticado com sepse, choque séptico e coagulação intravascular disseminada.^(1)^ Nessas circunstâncias, seria obrigatório realizar exame neurológico clínico e ressonância magnética do crânio É crucial determinar se a anisocoria que ocorreu 1 hora após a ressuscitação foi devido à hemorragia intracerebral ou se o paciente teve um acidente vascular cerebral isquêmico, sangramento subaracnóideo, hematoma subdural, hematoma epidural ou trombose de seios venosos. Uma vez que não foi possível alcançar saturação de oxigênio adequada após a segunda parada cardiorrespiratória 1 hora após a primeira parada cardiorrespiratória, deve-se descartar hipóxia cerebral.

Em segundo lugar, as hemoculturas também revelaram a presença de *H. influenzae*, que foi interpretada como uma coinfecção. Como os autores podem ter certeza de que a pneumonia, a sepse e a coagulação intravascular disseminada foram devidas à infecção por CA-MRSA, e não à infecção por *H. influenzae*? Em um estudo com 282 crianças com pneumonia necrosante, *H. influenzae* foi determinada como a causa em 28 pacientes.^(2)^

Em terceiro lugar, os achados da autópsia cerebral não foram relatados detalhadamente.^(1)^ É importante determinar se houve sinais de acidente vascular cerebral isquêmico, hemorragia intracerebral, hematoma subdural, hematoma epidural, sangramento subaracnóideo ou trombose de seios venosos. Além disso, sinais de encefalopatia séptica, meningite por MRSA, encefalite ou hipóxia cerebral cortical ou subcortical devem ser relatados.

Em quarto lugar, não foi descartada a possibilidade de infecção por coronavírus da síndrome respiratória aguda grave 2 (SARS-CoV-2). Como o caso ocorreu durante a pandemia, teria sido obrigatório relatar os resultados da transcrição reversa seguida de reação em cadeia da polimerase para SARS-CoV-2. É possível que, na verdade, a pneumonia tenha sido causada pelo SARS-CoV-2 e a CA-MRSA e *H. influenzae* tenham sido apenas superinfecções? Alguns casos de pneumonia necrosante causada por SARS-CoV-2 já foram relatados na literatura.^(3)^

Em quinto lugar, vários parâmetros de coagulação estavam claramente anormais. A Razão Normalizada Internacional foi de 3,76, o tempo de protrombina foi de 21% (n, > 70%), e o tempo de tromboplastina parcial ativada foi de 163 segundos (n, 29 - 38 segundos). Essas anormalidades foram devidas à coagulopatia de consumo, ou o paciente tinha história de distúrbio de coagulação? É possível que o sangramento intrapulmonar não tenha sido causado pela necrose tecidual, mas pelo distúrbio de coagulação presumivelmente adquirido?

Em resumo, o estudo tem várias limitações que complicam a interpretação dos resultados. Lidar com essas limitações poderia fortalecer e reforçar a afirmação do estudo. Antes que a pneumonia necrosante fatal possa ser atribuída à infecção por CA-MRSA, agentes infecciosos alternativos devem ser completamente excluídos, e complicações neurológicas requerem um extenso manejo diagnóstico.
